# Ceramide mediates FasL-induced caspase 8 activation in colon carcinoma cells to enhance FasL-induced cytotoxicity by tumor-specific cytotoxic T lymphocytes

**DOI:** 10.1038/srep30816

**Published:** 2016-08-04

**Authors:** Genevieve L. Coe, Priscilla S. Redd, Amy V. Paschall, Chunwan Lu, Lilly Gu, Houjian Cai, Thomas Albers, Iryna O. Lebedyeva, Kebin Liu

**Affiliations:** 1 Department of Biochemistry and Molecular Biology, Medical College of Georgia, Augusta, GA 30912, USA.; 2 Georgia Cancer Center, Augusta University, Augusta, GA 30912, USA; 3 Charlie Norwood VA Medical Center, Augusta, GA 30904, USA; 4 Department of Pharmaceutical & Biomedical Sciences, University of Georgia, Athens, GA 30602, USA; 5 Department of Chemistry and Physics, Augusta University, Augusta, GA 30912, USA

## Abstract

FasL-mediated cytotoxicity is one of the mechanisms that CTLs use to kill tumor cells. However, human colon carcinoma often deregulates the Fas signaling pathway to evade host cancer immune surveillance. We aimed at testing the hypothesis that novel ceramide analogs effectively modulate Fas function to sensitize colon carcinoma cells to FasL-induced apoptosis. We used rational design and synthesized twenty ceramide analogs as Fas function modulators. Five ceramide analogs, IG4, IG7, IG14, IG17, and IG19, exhibit low toxicity and potent activity in sensitization of human colon carcinoma cells to FasL-induced apoptosis. Functional deficiency of Fas limits both FasL and ceramide analogs in the induction of apoptosis. Ceramide enhances FasL-induced activation of the MAPK, NF-κB, and caspase 8 despite induction of potent tumor cell death. Finally, a sublethal dose of several ceramide analogs significantly increased CTL-mediated and FasL-induced apoptosis of colon carcinoma cells. We have therefore developed five novel ceramide analogs that act at a sublethal dose to enhance the efficacy of tumor-specific CTLs, and these ceramide analogs hold great promise for further development as adjunct agents in CTL-based colon cancer immunotherapy.

Fas, also termed CD95, APO1, or TNFRSF6, is a member of the tumor necrosis factor receptor superfamily. Fas exists as a trimeric membrane-bound surface receptor and is expressed on almost all types of cells throughout the mammalian body^1^. In contrast, the expression of the physiological ligand of Fas, Fas ligand (FasL, CD95L, or TNFSF6), is restricted to highly selective types of cells, primarily to activated T cells, NKT cells, and NK cells^2^,^3^. Expression of FasL on certain non-lymphoid tissues, such as the eye and testis, has been reported but both its expression and function are still controversial^4^. FasL has also been reported to be expressed in certain tumor cells, mainly as soluble FasL^5^,^6^,^7^. The expression and function of soluble FasL in tumor cells are hotly debated^8^. However, it is generally believed that only the membrane-bound form of FasL is capable of inducing apoptosis^9^.

Engagement of the Fas receptor by soluble FasL has been shown to initiate a non-apoptotic survival signal^10^,^11^,^12^,^13^. However, the first and best-characterized function of Fas is its ability to mediate apoptosis in various types of cells, ranging from the so called type 1 lymphocytes to type 2 hepatocytes and epithelial tumor cells^1^,^14^,^15^,^16^. Fas is highly expressed in normal human colon epithelial cells. It has been shown that Fas protein level is down-regulated in primary human colon carcinoma and complete loss of Fas expression often occurs in metastatic human colon carcinoma^17^. It is known that FasL of cytotoxic T lymphocytes (CTLs) plays an essential role in suppression of spontaneous tumor development^18^,^19^,^20^,^21^. Therefore, human colon carcinoma may use down-regulation of Fas expression as a mechanism to escape host cancer immune surveillance. Therapeutic means to upregulate Fas expression level may be an effective way to suppress human colon carcinoma immune evasion. Because Fas receptor clustering and oligomerization is essential for Fas function^22^,^23^,^24^,^25^, alternatively, therapeutic means to enhance Fas activation and resultant caspase 8 activation may represent another effective approach to suppress human colon carcinoma immune escape.

Ceramide, the central metabolite of the sphingolipid metabolism pathway, is a key secondary messenger that mediates multiple cellular functions, including cell proliferation, apoptosis, motility, differentiation, stress responses, protein synthesis, carbohydrate metabolism, immunity, and angiogenesis^26^,^27^,^28^,^29^. Compelling experimental data from mouse models and human patients have shown that ceramide deregulation is a key factor in tumor progression and cancer cell resistance to chemotherapeutic agents and radiation^30^,^31^. The crucial role of ceramide in tumor development and cancer cell responses to chemotherapy and radiation have led to extensive studies to target the ceramide metabolism pathways for development of potential anticancer therapies. For the last two decades, extensive efforts have been devoted to develop ceramide analogs to mimic natural ceramide, and numerous ceramide analogs with different chemical and biological properties have been developed^32^,^33^,^34^. However, these ceramide analogs are primarily designed for their direct anti-cancer activity.

Although trimerized Fas can initiate apoptosis, it seems that super-aggregation of trimerized Fas may enhance FasL-induced apoptosis via a ceramide-dependent mechanism in both type 1 and type 2 cells^22^,^35^,^36^,^37^,^38^,^39^,^40^. As such, ceramide analogs have the potential to enhance Fas receptor aggregation and thus increase the efficacy of FasL-induced apoptosis. However, this is an area that has been largely unexplored^41^. We hypothesized that ceramide analogs may enhance Fas aggregation to increase Fas receptor affinity to FasL, and thereby sensitize colon carcinoma cells to FasL-induced apoptosis. We synthesized twenty ceramide analogs based on structure and functional relationship and identified five novel ceramide analogs that exhibit low toxicity yet effectively increase colon carcinoma cell sensitivity to FasL-induced apoptosis of tumor-specific CTLs. These five ceramide analogs thus have the great potential to be developed as adjunct agents to enhance the efficacy of colon cancer immunotherapy.

## Results

### Fas protein level decreases as cancer progresses in human colon carcinoma

**Figure 1:**
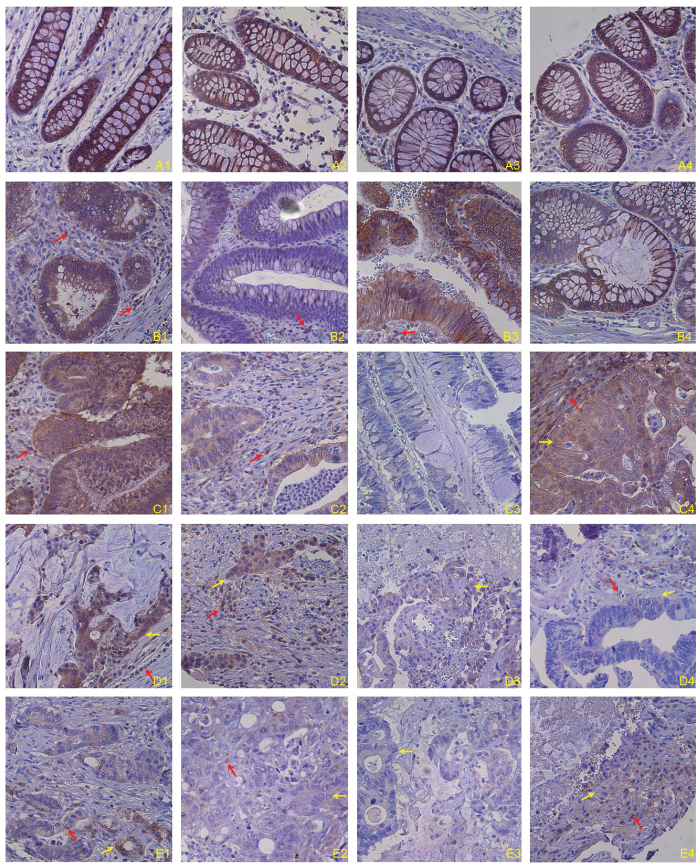
Fas protein level in normal human colon and human colorectal carcinoma tissues. Human colon carcinoma tissues were stained with anti-human Fas monoclonal antibody. Brown color indicates Fas protein level, with counterstaining by hematoxylin in blue. Shown are representative images of adjacent normal human colon tissues from colon cancer patients (A1–4 indicates tissues from four patients), adenomas (B1–4), primary invasive adenocarcinoma (C1–4), colorectal adenocarcinoma metastatic to lymph nodes (D1–4), and colorectal adenocarcinoma metastatic to liver (E1–4).

To determine Fas protein levels in normal colonic epithelial and colon carcinoma cells, adjacent normal colon tissues from human colon cancer patients were stained with human Fas-specific antibody by immunohistochemical methods. Fas protein level is high in all five normal colon tissues from five colon cancer patients (Fig. 1A and Table S1). Fas protein level in nine of the fourteen adenomas analyzed is as high as in normal colon tissues. The remaining five specimens showed medium levels of Fas protein (Fig. 1B and Table S1). For the fourteen adenocarcinoma specimens analyzed, Fas protein levels range from high to low. Approximately 35.5% of specimens are high in Fas, 29% have medium Fas protein level, whereas about 35.5% exhibit low to undetectable Fas protein levels (Fig. 1C and Table S1). Among the five lymph node (LN) metastatic specimens analyzed, Fas protein levels showed similar patterns as the adenocarcinomas (Fig. 1D and Table S1). Fas protein level is lower overall in the liver metastatic specimens, with six of the seven specimens exhibiting low to undetectable Fas protein level and only one liver metastasis showing medium level of Fas protein (Fig. 1E and Table S1). Overall, our data indicates that Fas protein level decreases as colon cancer progresses.

### Fas receptor is expressed on human colon carcinoma cell surface

The above observations suggest that as the cancer progresses to advanced stages, colon carcinoma cells may progressively down-regulate Fas expression to decrease cell sensitivity to FasL. It is the Fas receptor expressed on the tumor cell surface that mediates FasL-induced apoptosis. Next, we analyzed Fas protein levels on human colon carcinoma cell surfaces. Among the six human colon carcinoma cell lines examined, Fas protein is detected in five cell lines (Fig. S1A). The Fas receptor levels are high in two cell lines (SW480 and LS174T), medium in three cell lines (HCT116, HT29, and RKO), and undetectable in CACO2 cells (Fig. S1B). These observations indicate that Fas receptor is expressed in the majority of human carcinoma cell lines.

### Fas receptor level is not correlated with human carcinoma cell sensitivity to FasL-induced apoptosis.

**Figure 2:**
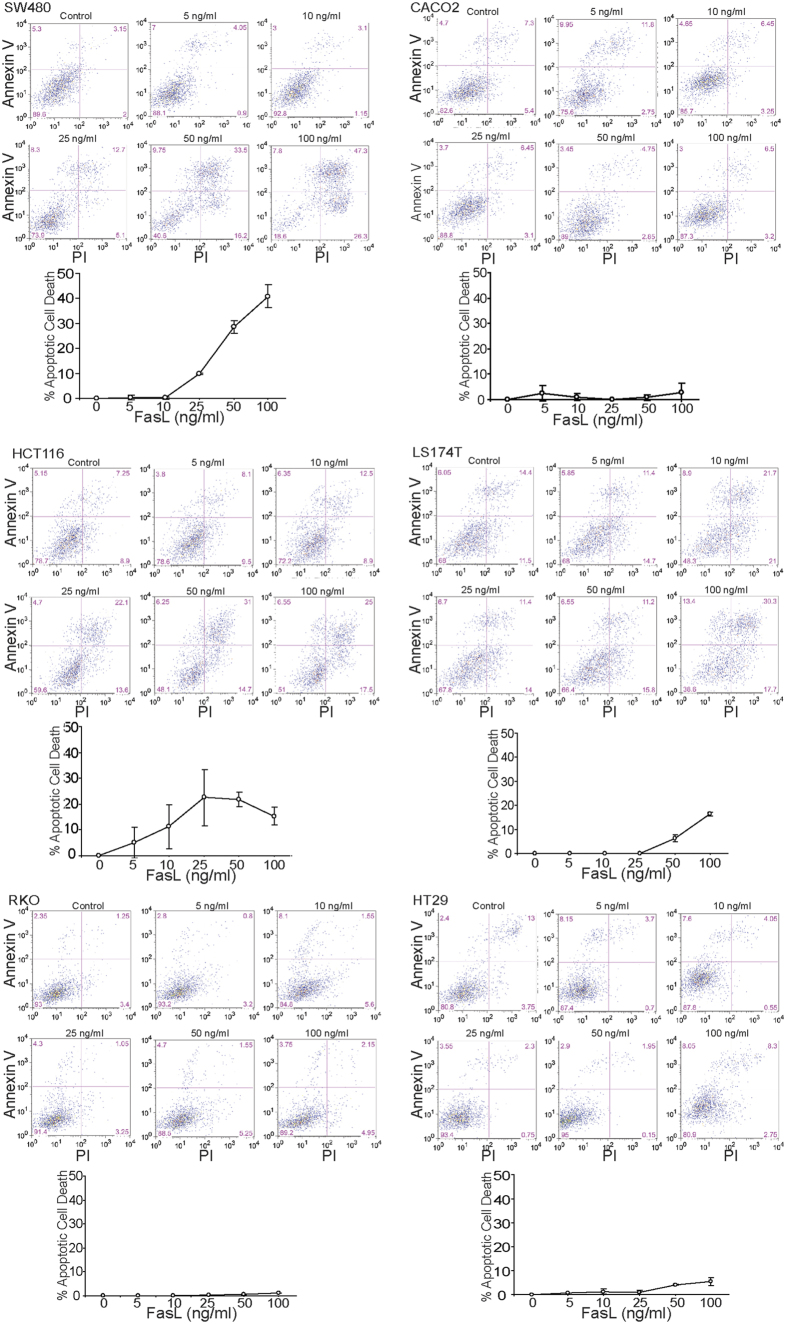
Sensitivity of human colon carcinoma cells to FasL-induced apoptosis. The indicated human colon carcinoma cells were cultured in the presence of MegaFasL at the indicated concentrations for approximately 24 h. Both floating and adherent cells were harvested and stained for Annexin V and PI. Cells were analyzed by flow cytometry. For each cell line, the top panel shows representative plots of apoptotic cell death. The bottom panel shows quantification of apoptotic cell death. Percent apoptotic cell death was calculated as (% Annexin V^+^PI^+^ cells in the presence of FasL) − (% Annexin V^+^PI^+^ cells in the absence of FasL). Column: mean; Bar:SD.

To determine whether the Fas receptor level is associated with sensitivity of these human colon carcinoma cells to FasL-induced apoptosis, human colon carcinoma cells were treated with various doses of FasL and analyzed for apoptotic cell death. SW480 cells express high levels of Fas receptor and are sensitive to FasL-induced apoptosis (Fig. 2). HCT116 cells express medium levels of Fas receptor and are as sensitive to FasL-induced apoptosis as SW480 cells. However, LS174T cells exhibit the highest Fas receptor levels among the six cell lines, but are less sensitive to FasL-induced apoptosis as compared to SW480 and HCT116. RKO and HT29 cells express medium levels of Fas receptor and are not sensitive to FasL-induced apoptosis (Fig. 2). These observations thus indicate that the majority of human colon carcinoma cells have detectable Fas receptor on their surface, and Fas^+^ human colon carcinoma cells are not necessarily sensitive to FasL-induced apoptosis.

### Development of ceramide analogs for sensitization of Fas-mediated apoptosis

We have analyzed the structures and functions of ceramide analogs^31^,^41^ and synthesized twenty ceramide analogs to be developed as drug that can sensitize colon carcinoma cells to FasL-mediated apoptosis (Table S2). We first tested the cytotoxicity of these twenty ceramide analogs using SW480 cells. These ceramide analogs have an IC_50_ ranging from about 5 to 50 μM (Fig. S2). Two of the analogs (IG10 and IG20) exhibited no cytotoxicity at the doses tested (Fig. S2).

### Ceramide analogs sensitize human colon carcinoma cells to FasL-induced apoptosis

**Figure 3:**
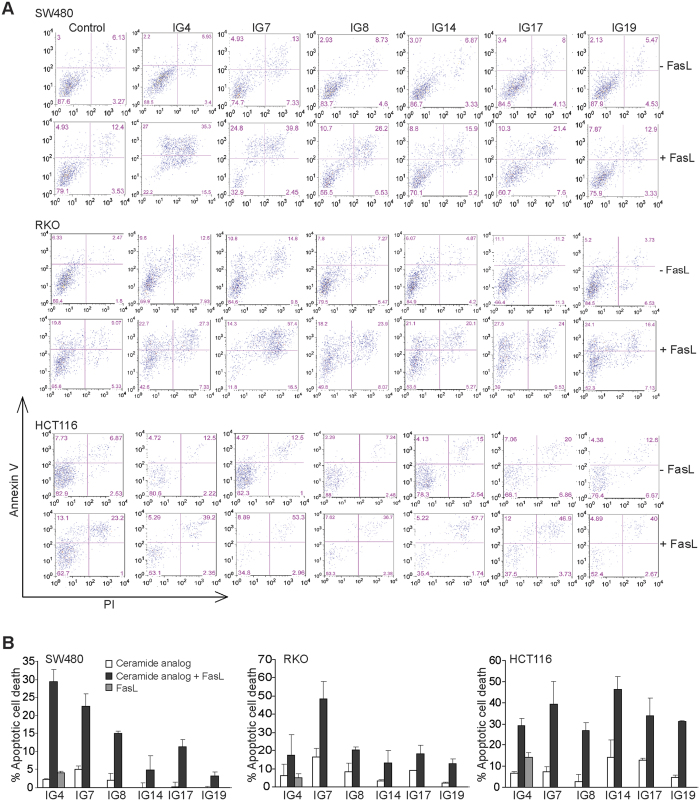
Identification of novel ceramide analogs that sensitize human colon carcinoma cells to FasL-induced apoptosis. (**A**) Human colon carcinoma SW480, RKO and HCT116 cells were cultured in the presence of the indicated ceramide analogs (10 μM), with or without MegaFasL (SW480 = 25 ng/ml, RKO = 50 ng/ml, and HCT116 = 10 ng/ml) for approximately 24 h. Both floating and adherent cells were harvested, stained with Annexin V and PI, and analyzed by flow cytometry. Shown are representative plots of apoptotic cell death. (**B**) Quantification of apoptotic cell death. % apoptotic cell death was calculated as % Annexin V^+^PI^+^ cells in the presence of ceramide analogs plus MegaFasL - % Annexin V^+^PI^+^ cells in the control group. Column: mean; Bar: SD.

Next, we tested the efficacy of sublethal doses of these ceramide analogs at enhancing FasL-induced apoptosis using SW480, RKO, and HCT116 cell lines. Tumor cells were treated with a sublethal dose of each of these twenty ceramide analogs alone or in combination with FasL, and analyzed for apoptosis. Among the twenty ceramide analogs, six analogs (IG4, IG7, IG8, IG14, IG17, and IG19) effectively increased the sensitivity three human colon carcinoma cell lines to FasL-induced apoptosis (Fig. 3AB). Red arrows indicate immune cells and yellow arrows indicate tumor cells.

### Ceramide analogs increase FasL-induced caspase 8 activation

**Figure 4:**
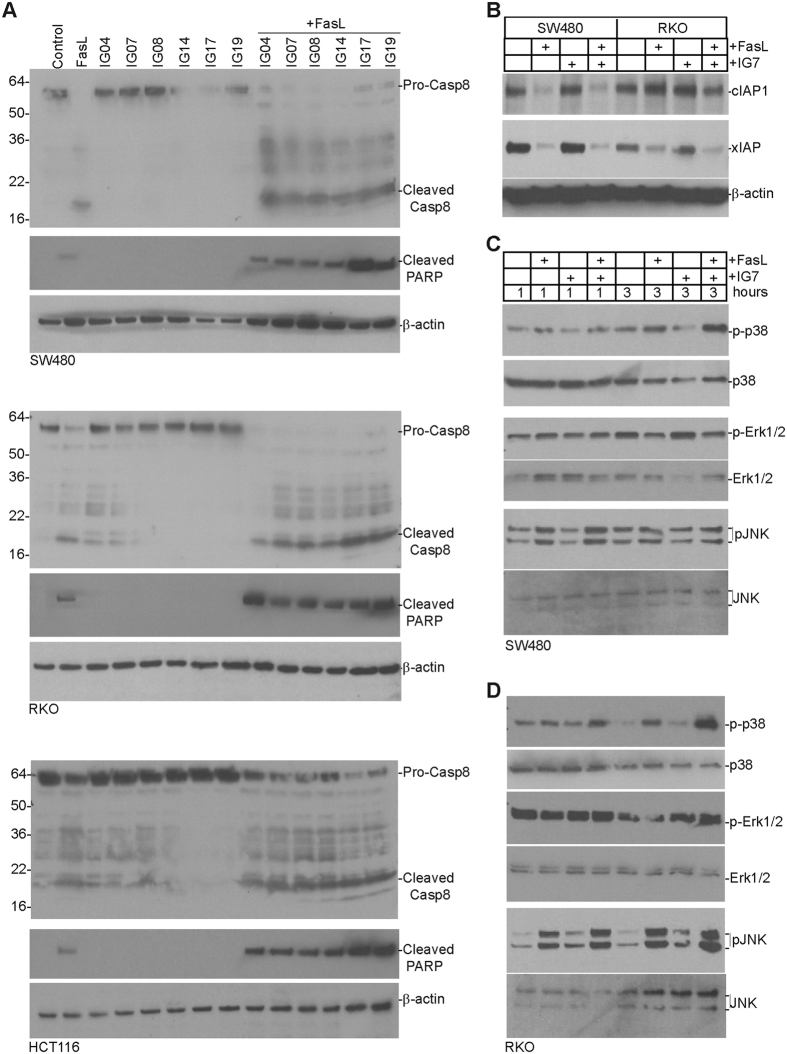
Fas mediates activation of caspase 8 and MAPK and ceramide analogs enhance FasL-induced caspase 8 activation. (**A**) Human colon carcinoma SW480, RKO, and HCT116 cells were cultured in the presence of the indicated ceramide analogs or ceramide analogs plus MegaFasL for 4 h. Cells were collected and lysed in cytosol buffer. Cytosolic fractions were resolved in 4–20% SDS polyacrylamide gel and analyzed by Western blotting using anti-active caspase 8 and anti-cleaved PARP antibodies, respectively. The membranes were stripped and re-probed with anti-β-actin antibody. The pro-caspase 8, cleaved caspase 8, cleaved PARP and β-actin are indicated at the right. The locations of molecular weight markers are indicated at the left. (**B**) SW480 and RKO cells were cultured in the presence of FasL (100 ng/ml), IG7 (10 μM), or both FasL and IG7 for 24 hours and total lysate was prepared from the treated cells. Total lysate was analyzed by Western blotting as in A with antibodies that are specific for the indicated proteins. (**C**,**D**) SW480 (**C**) and RKO (**D**) cells were cultured in the presence of FasL (50 ng/ml), IG7 (10 μM) or both FasL and IG7 for 1 and 3 hours. Cytosol fractions were isolated as in A and analyzed by Western blotting as in A with antibodies that are specific for the indicated proteins.

FasL binding to the Fas receptor induces DISC formation and subsequent caspase 8 activation that initiates the Fas-mediated apoptosis pathway^1^. We then aimed at testing the hypothesis that these ceramide analogs modulate caspase 8 activation to increase human colon carcinoma cell sensitivity to FasL-induced apoptosis. Tumor cells were treated with either FasL, ceramide analogs, or ceramide analogs in combination with FasL and analyzed for caspase 8 activation. Western blotting analysis indicated that FasL induces caspase 8 activation as evidenced by degradation of procaspase 8 and generation of cleaved caspase 8 in SW480, RKO, and HCT116 cells (Fig. 4A). None of the six ceramide analogs at their sublethal doses induces caspase 8 activation. However, combination of a ceramide analog with FasL increased procaspase 8 degradation and generation of active caspase 8 in all three human colon carcinoma cell lines tested (Fig. 4A). Furthermore, the cleavage of PARP, a biochemical indicator of apoptosis, was also enhanced by all six ceramide analogs (Fig. 4A).

Next, we sought to determine whether these ceramide analogs decrease xIAP and cIAP1 protein level as previously demonstrated with another ceramide analog^41^. Because these six analogs act similarly in terms of caspase 8 activation (Fig. 4A), we used IG7 as a representative ceramide analog for the following studies. The FasL-sensitive SW480 and FasL-resistant RKO cells were treated with FasL and IG7, either alone or in combination. Western blotting analysis revealed that FasL decreased cIAP1 protein level in SW480 cells but not in RKO cells (Fig. 4B). FasL decreased xIAP protein level in both SW480 and RKO cells (Fig. 4B). However, ceramide analog IG7 did not alter cIAP1 and xIAP protein levels in SW480 and RKO cells (Fig. 4B).

### Ceramide enhances FasL-induced apoptosis in a Fas receptor-dependent manner

**Figure 5:**
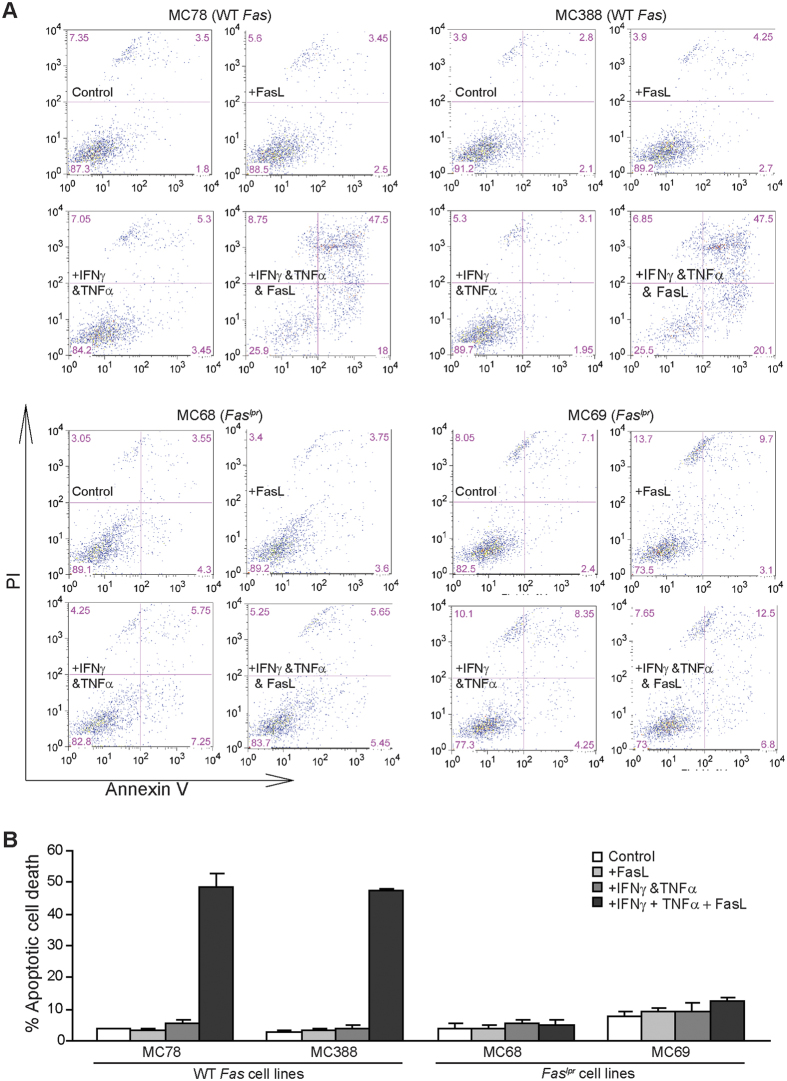
Fas receptor is essential for FasL-induced apoptosis. (**A**) Tumor cells were cultured in the presence of FasL (100 ng/ml), IFNγ (100 U/ml) + TNFα (100 U/ml), or IFNγ (100 U/ml) + TNFα (100 U/ml) + FasL for approximately 24 h. Both floating and adherent cells were collected and stained with PI and Annexin V. Cells were then analyzed by flow cytometry. (**B**) Cells as shown in A are quantified for apoptosis. Percent apoptotic cell death was calculated as (% Annexin V^+^PI^+^ cells of treated cells) − (% Annexin V^+^PI^+^ cells in the absence of FasL). Column: mean; Bar: SD.

Our above observations indicate that these ceramide analogs enhance FasL-induced apoptosis in tumor cells (Fig. 3). To determine whether ceramide analog-enhanced FasL function depends on the Fas receptor, we made use of two pairs of WT and Fas receptor-deficient tumor cell lines and analyzed their sensitivity to FasL-induced apoptosis. MC78 and MC388 are two sarcoma cell lines established from WT C57BL/6 mice, and MC68 and MC69 are two sarcoma cell lines established from *fas*^*lpr*^ mice^42^. These four cell lines are resistant to FasL. IFNγ and TNFα treatment sensitized MC78 and MC388 to FasL-induced apoptosis (Fig. 5A). However, MC68 and MC69 cells are completely resistant to FasL even after IFNγ and TNFα treatment (Fig. 5A,B). These observations determine that Fas receptor is essential for FasL-induced apoptosis in tumor cells.

**Figure 6:**
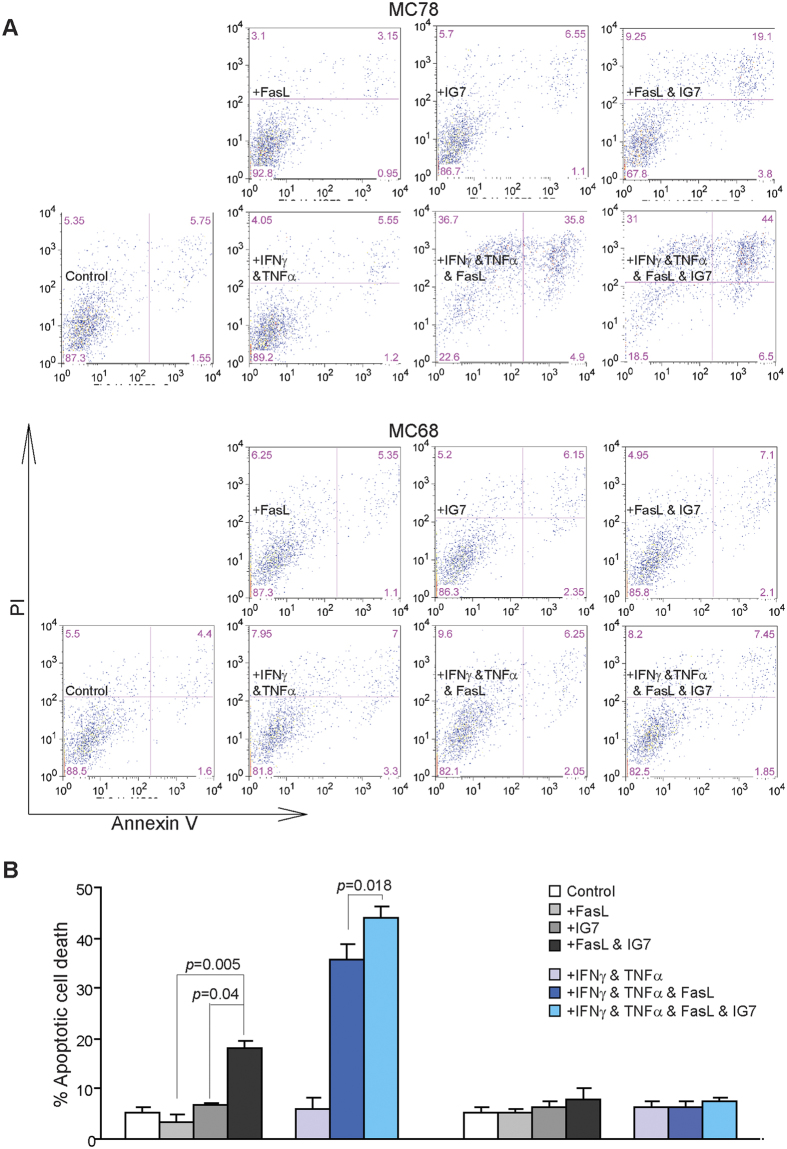
Ceramide analog enhances FasL-induced apoptosis in tumor cells in a Fas-dependent mechanism. (**A**) Tumor cells were treated as shown in the absence or presence of ceramide analog IG7 (10 μM) for approximately 24 h. Both floating and adherent cells were collected and stained with PI and Annexin V. Cells were then analyzed by flow cytometry. (**B**) Cells as shown in A are quantified for apoptosis. Percent apoptotic cell death was calculated as (% Annexin V^+^PI^+^ cells of treated cells) − (% Annexin V^+^PI^+^ cells in the absence of FasL). Column: mean; Bar: SD.

Next, MC78 and MC68 cells were sensitized with IFNγ and TNFα, and treated with FasL and a sublethal dose of IG7, either alone or in combination. Analysis of tumor cell apoptosis shows that IG7 enhances FasL-induced apoptosis in the WT MC78 cells (Fig. 6A,B). However, IG7 function in enhancement of FasL-induced apoptosis is abolished in the Fas-deficient MC68 tumor cells (Fig. 6B). These observations thereby indicate that ceramide analog function in enhancement of FasL-induced apoptosis depends on Fas receptor in the tumor cells.

### Ceramide analogs enhances FasL-induced activation of the MAPK signaling pathway and NF-κB

**Figure 7:**
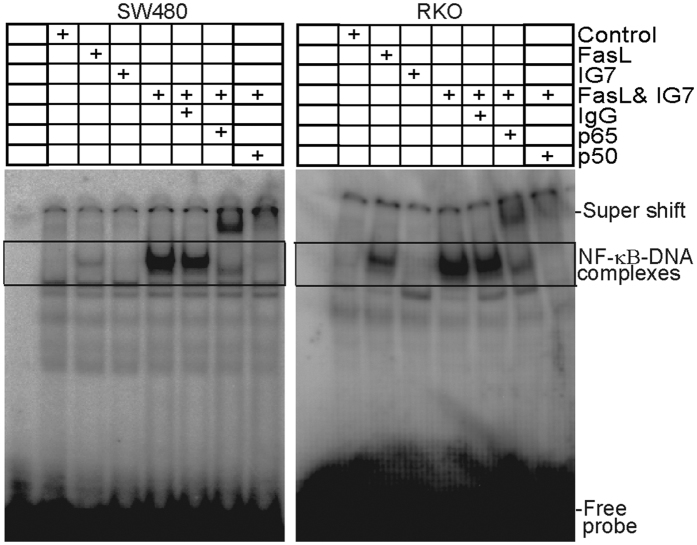
FasL induces NF-κB activation and ceramide analog increases FasL-induced NF-κB activation. SW480 and RKO cells were treated with FasL (50 ng/ml), IG7 (10 μM), or FasL and IG7 for 1h and nuclear extracts were prepared from the tumor cells and analyzed for canonical NF-κB activity using EMSA with NF-κB consensus sequence-containing DNA probe as described in the materials and methods. Anti-p65 and anti-p50 antibodies were used to identify the canonical NF-κB-DNA complexes. The DNA-NF-κB complexes are indicated at the right.

In addition to activation of the apoptosis signaling pathway, Fas signaling also activates the MAPK signaling pathway and NF-κB^10^,^11^,^12^,^13^,^42^. It has also been reported that caspase 8 activation is essential for Fas-mediated MAPK activation^13^. To determine whether ceramide analogs regulate FasL-induced activation of MAPK and NF-κB in human colon carcinoma cells, SW480 and RKO cells were treated with FasL and ceramide analog IG7. Western blotting analysis indicated that FasL also induces phosphorylation of p38, Erk1/2, and JNK in both SW480 and RKO cells (Fig. 4C,D). Additionally, IG7 enhances FasL-induced phosphorylation of p38 and JNK (Fig. 4C,D). FasL and IG7 exhibit no effects on Erk activation (Fig. 4C,D). Analysis of NF-κB DNA binding activity indicates that FasL induces activation of the canonical p65/p50 heterodimer NF-κB in both SW480 and RKO cells. Interestingly, the ceramide analog dramatically increases NF-κB activation in both SW480 and RKO cells (Fig. 7).

### Ceramide analogs effectively enhance human colon carcinoma cell lysis through FasL on tumor-specific CTLs

**Figure 8:**
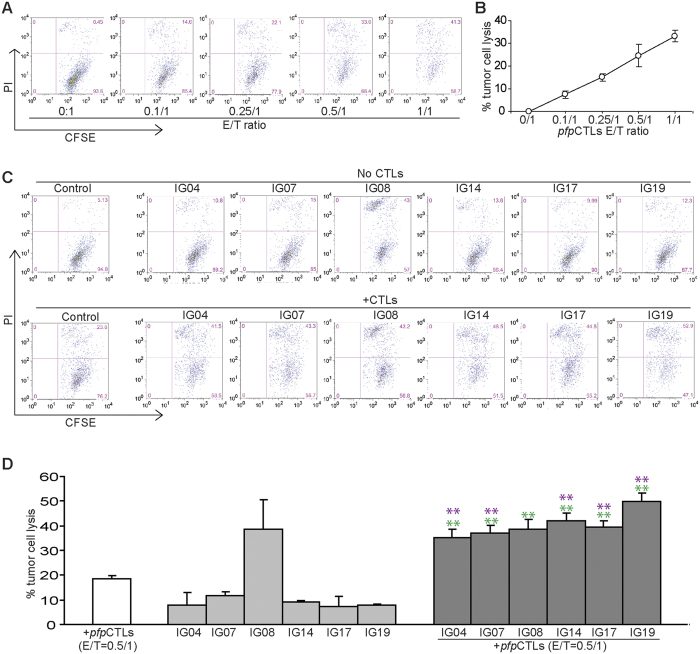
Identification of novel ceramide analogs that increase efficacy of tumor-specific CTL-mediated tumor lysis. (**A**) Mouse colon carcinoma CT26 cells were labeled with CFSE and seeded in U-bottom 96-well plates. Tumor-specific perforin-deficient *pfp*CTLs were then added to the tumor cultures at the indicated effector/tumor ratios (E/T ratios) and cultured for approximately 24 h. CTL and tumor culture mixtures were harvested and stained with PI and analyzed by flow cytometry. Shown are representative plots. (**B**) Quantification of CTL-induced tumor cell death kinetics. Cells as shown in A were gated for CFSE^+^ tumor cells. The gated cells were then analyzed for PI^+^ cells. % tumor cell lysis was calculated as % CFSE^+^PI^+^ cells in the presence of CTLs - % CFSE^+^PI^+^ cells in the absence of CTLs. (**C**) CT26 cells were labeled with CFSE as in A and cultured in the presence of the indicated ceramide analogs (10 μM) without (top panel) or with (bottom panel) pfpCTLs for approximately 24 h. CTL-induced tumor cell death was analyzed as in A. (**D**) Quantification of CTL-induced tumor cell death in the absence or presence of ceramide analogs. Cells as shown in C were gated for CFSE^+^ tumor cells. The gated cells were then analyzed for PI^+^ cells. % tumor cell lysis was calculated as % CFSE^+^PI^+^ cells in the presence of ceramide analogs or ceramide analogs plus *pfp*CTLs - % CFSE^+^PI^+^ cells in the absence of ceramide analogs or ceramide analogs plus *pfp*CTLs. Column: mean; Bar: SD. Red **indicated p < 0.01 between ceramide analog + *pfp*CTLs group and pfpCTLs only group, and green **indicates p < 0.01 between ceramide analog + *pfp*CTLs group and ceramide analog only group.

FasL on CTLs plays an essential role in host cancer immunosurveillance to suppress spontaneous cancer development^18^,^19^,^43^. To determine whether the observation that these six ceramide analogs can sensitize FasL-induced apoptosis can be extended to CTL-mediated tumor lysis, we performed a proof of principle study. A perforin-deficient CTL line (*pfp*CTL) that recognizes mouse colon carcinoma cell line CT26 was used to determine whether these six ceramide analogs are effective in sensitizing CT26 tumor cells to FasL-mediated cytotoxicity of tumor-specific *pfp*CTLs. CT26 is a mouse colon carcinoma cell line that expresses low levels of Fas (Fig. S3A). Fas protein level on CT26 cell surface can be increased by IFNγ and TNFα, two cytokines that are produced by activated T cells (Fig. S3B). As expected, *pfp*CTLs kill CT26 cells in a dose-dependent manner (Fig. 8A,B). Addition of sublethal doses of ceramide analogs significantly increased the efficacy of *pfp*CTL-mediated lysis of CT26 tumor cells (Fig. 8C,D). One of the ceramide analogs, IG8, exhibits high cytotoxicity to CT26 tumor cells *in vitro*. The other five ceramide analogs exhibited low cytotoxicity at the dose used *in vitro* but showed dramatic efficacy in enhancement of the tumor-specific CTL activity in lysis of CT26 tumor cells. Initial *in vivo* toxicity analysis revealed that IG8 is toxic in tumor-bearing mice and all other five ceramide analogs exhibit no apparent toxicity at a dose as high as 100 mg/kg body weight (Fig. S4). Taken together, we have developed five ceramide analogs that exhibit no apparent toxicity at a dose as high as 100 mg/kg body weight but high efficacy as adjunct agents in enhancement of the FasL-mediated effector mechanism of tumor-specific CTLs.

## Discussion

Fas expression diminishes in human colon tumor cells as the cancer progresses. Because Fas is the death receptor that mediates FasL-induced apoptosis, and it has been shown that FasL on CTLs plays an essential role in suppression of spontaneous tumor development^18^,^19^,^43^, it therefore seems that human colon carcinoma cells might use down-regulation of Fas as a mechanism to escape the host cancer immune surveillance to progress and metastasize. One potentially effective approach to suppress colon cancer progression is to restore Fas expression. However, we show here that the majority of human colon carcinoma cells, particularly the primary colon carcinoma cells, still express Fas protein. Furthermore, we have also shown that the level of Fas expression is not necessarily correlated with the tumor cell sensitivity to FasL-induced apoptosis since Fas oligomerization—and subsequently caspase 8 activation—plays a critical role in FasL-induced apoptosis^22^,^23^,^24^,^25^. Therefore, therapeutic means to enhance Fas oligomerization to increase caspase 8 activation may represent an effective approach to increase colon carcinoma cell sensitivity to FasL-induced apoptosis to suppress colon carcinoma cell immune evasion. We have developed and functionally characterized five ceramide analogs that can effectively increase human colon carcinoma cell sensitivity to FasL-induced apoptosis at sublethal doses. Our previous study showed that the ceramide analog LCL85 is also effective in sensitizing colon carcinoma cells to FasL-induced apoptosis *in vitro* and suppressing tumor growth *in vivo*^41^. Tumor-bearing mice tolerate LCL85 up to 5 mg/kg body weight. However, our initial *in vivo* toxicity study showed that IG4, IG7, IG14, IG17, and IG19 exhibit no apparent toxicity at a dose as high as 100 mg/kg body weight in tumor-bearing mice. These ceramide analogs are non-toxic and effectively increase colon carcinoma cell sensitivity to FasL-mediated cytotoxicity by tumor-specific CTLs *in vivo*.

CTL-based cancer immunotherapies, including CTL adoptive transfer, check point blockade (anti-PD-1 and anti-CTLA4 mAb), and CAR T cell immunotherapy have recently shown remarkable and durable efficacy in suppression of various human cancers in the clinics^44^,^45^,^46^. However, the patient objective response rate for anti-PD-1 immunotherapy is only about 6–17%^46^. All of these immunotherapies depend on CTL-induced target tumor cell apoptosis. Apoptosis resistance of cancer cells, either intrinsic or acquired, is a hallmark of human cancer^47^. Consequently, if cancer cells are not sensitive to apoptosis, regardless of how potent the CTLs are, the target tumor cell lysis efficacy of immunotherapy is not going to be high. It is known that CTLs kill target cells primarily through two effector mechanisms: the perforin-mediated and Fas-mediated cytotoxicity^48^. We have developed five novel ceramide analogs that are potentially safe and can significantly increase colon carcinoma cell sensitivity to FasL-induced apoptosis of tumor-specific CTLs. These ceramide analogs may thus have the potential to be translated as adjunct agents to increase the efficacy of CTL adoptive transfer, check point blockade, and CAR T cell immunotherapy.

In addition to cancer cell apoptosis resistance, immune suppression is another major impediment in CTL-based cancer immunotherapy^49^. Although antigen-specific CTLs use both perforin-mediated and FasL-mediated cytotoxicity to kill target tumor cells under physiological conditions^19^,^48^, recent studies showed that the immune suppressive Treg cells selectively inhibit the perforin-mediated cytotoxicity without affecting T cell activation^50^,^51^. Therefore, the FasL-mediated cytotoxicity of tumor-specific CTLs should still be active in the immune suppressive tumor microenvironment. Our finding that our newly developed ceramide analogs effectively increase the efficacy of FasL-mediated target colon cancer cell lysis by tumor-specific CTLs suggests that these ceramide analogs may have the potential to increase CTL efficacy against immune suppressive cancers.

It is well-documented that ceramide mediates the expression of apoptosis-regulatory genes and apoptosis pathways^28^,^34^,^41^,^52^,^53^,^54^,^55^,^56^,^57^,^58^,^59^. Ceramide has also been shown to regulate Bcl-x alternative splicing to decrease Bcl-xL levels^54^ and mediates Bak, Bax, and Bcl-2 functions in the intrinsic apoptosis pathway^52^,^58^,^60^,^61^,^62^,^63^. Ceramide also regulates xIAP and cIAP1 protein levels to mediate apoptosis^41^,^59^,^64^,^65^. We show here that although our newly developed ceramide analogs enhance FasL-induced caspase 8 activation and increase FasL-induced apoptosis in human colon carcinoma cells, ceramide analog IG7 apparently does not alter cIAP1 and xIAP protein levels in human colon carcinoma cells, suggesting that IG7 acts through a different mechanism from LCL85 in enhancing FasL-induced apoptosis. The role of these ceramide analogs in regulating other apoptosis regulatory genes remains to be determined. It is also known that ceramide mediates the expression of genes involved in tumor cell progression such as MMPs^27^,^28^. It is possible that these ceramide analogs may mediate the expression of apoptosis and tumor progression regulatory genes in human colon carcinoma cells, which remains to be determined. In addition, it has been well-established that Fas receptor also mediates non-apoptotic and cell survival signaling pathways^10^,^11^,^12^,^13^. Indeed, we observed that FasL-induces activation of MAPK in human colon carcinoma cells. Furthermore, FasL induces NF-κB activation and the ceramide analog IG7 enhances FasL-induced NF-κB activation. However, despite FasL activation of these signaling pathways that have contrasting functions in cell survival and death, the final consequence of FasL and ceramide analog treatment is tumor cell death. The simultaneous activation of MAPK, NF-κB, and caspase 8 and their respective functions in colon carcinoma cells remains to be determined. Nevertheless, our observation that these ceramide analogs enhance FasL-induced caspase 8 activation suggests that these ceramide analogs effectively mediate the Fas receptor DISC complex conformation to increase colon cancer cell sensitivity to apoptosis induction by T cells, which provides the molecular mechanism and strong rationale for further development of these ceramide analogs as adjunct agents in cancer immunotherapy.

## Methods

### Human colon cancer cells and mice

Human colon cancer cell lines SW480, LS174T, HCT116, HT29, RKO, and CACO2 were obtained from American Type Culture Collection (ATCC) (Manassas, VA). ATCC has characterized these cells by morphology, immunology, DNA fingerprint, and cytogenetics. All cells are cultured in RPMI medium plus 10% fetal bovine serum. MC78 and MC388 are sarcoma cell lines established from WT C57BL/6 mice, and MC68 and MC69 are sarcoma cell lines established from *fas*^*lpr*^ mice as previously described^42^. BALB/c mice were obtained from Charles River Laboratories. All mouse studies were performed according to a protocol approved by Augusta University Institutional Animal Care and Use Committee. All experiment methods were performed in accordance with the relevant guidelines established by Augusta University Institutional Biosafety Committee.

### Cell viability assays

Ceramide analogs were dissolved in DMSO to make stock solution and diluted with medium for *in vitro* cell treatment. Cells were seeded in 96-well plates at 2 × 10^3^ cells/well in 100 μl culture medium for three days. Cell viability assays were performed using the MTT cell proliferation assay kit (ATCC, Manassas, VA) according to the manufacturer’s instructions.

### Flow cytometry

Cells were stained with fluorescent dye-conjugated anti-human Fas (Clone: DX2, Biolegend, San Diego, CA). Cells were then analyzed by flow cytometry.

### Immunohistochemistry

Human colon cancer tissue microarray slides were provided by the Cooperative Human Tissue Network (Mid-Atlantic Division, University of Virginia, Charlottesville, VA). The tissues were stained with anti-human Fas (Clone: B-10, Santa Cruz Biotech, Dallas, TX). Slides were counterstained with hematoxylin (Richard-Allan Scientific, Kalamazoo, MI). Immunohistochemical staining was performed at the Georgia Pathology Services.

### Tumor cell apoptosis assay

Tumor cells were cultured in the presence of MegaFasL at the indicated concentrations as previously described^66^. FasL (Mega-Fas Ligand^®^, kindly provided by Drs. Steven Butcher and Lars Damstrup at Topotarget A/S, Denmark) is a recombinant fusion protein that consists of three human FasL extracellular domains linked to a protein backbone comprising the dimer-forming collagen domain of human adiponectin. The Mega-Fas Ligand was produced as a glycoprotein in mammalian cells using Good Manufacturing Practice compliant process in Topotarget A/S (Copenhagen, Denmark). IFNγ and TNFα proteins were obtained from R&D Systems Inc. (Minneapolis, MN). For tumor cell apoptosis analysis, cells were stained with Alexa Fluor 647 Annexin V (Biolegend) in Annexin V-binding buffer (10 mM Hepes, pH 7.4, 140 mM NaCl, 2.5 mM CaCl2) for 30 min at 4 °C. Propidium Iodide was then added to the cell suspension, and cells were analyzed by flow cytometry as previously described^67^.

### Electrophoresis Mobility Shift Assay (EMSA) of NF-κB activation

NF-κB activation was analyzed using NF-κB probe (AGT TGA GGG GAC TTT CCC AGG C, Santa Cruz Biotech) as previously described^68^. Briefly, the end-labeled probes were incubated with nuclear extracts for 20 min at room temperature. Anti-p65 and p50 antibodies (Santa Cruz Biotech) were included to identify specific canonical NF-κB-DNA complexes. DNA-protein complexes were separated by electrophoresis in 6% polyacrylamide gels and identified using a phosphoimage screen (Molecular Dynamics) and the images were acquired using a Strom 860 imager (Molecular Dynamics).

### Western blotting analysis

Western blotting analysis was performed as previously described^69^. Briefly, tumor cells were cultured in the presence of the indicated ceramide analogs or ceramide analogs plus MegaFasL for 4 h. Cells were collected and lysed in cytosol buffer [10 mM Hepes, pH 7.4, 250 mM Sucrose, 70 mM KCl, 1.5 mM MgCl2, 1 mM EDTA, 1 mM EGTA, protease and phosphatase inhibitor cocktails (Calbiochem, Billerica, MA), and 0.01% digitonin] for 10 min. Cytosolic fractions were resolved in 4–20% SDS polyacrylamide gel and analyzed by Western blotting. Anti-cleaved caspase 8 and anti-cIAP1 were obtained from R&D Systems. Anti-cleaved human PARP, anti-xIAP, anti-p38, anti-p-p38, anti-pErk1/2, anti-Erk1/2, anti-pJNK, and anti-JNK antibodies obtained from Cell Signaling. β-actin was obtained from Sigma-Aldrich.

### CTL Cytotoxicity Assays

Perforin-deficient CTLs were generated and maintained by weekly stimulation with AH1 peptide as previously described^21^. CT26 cells were labeled with CellTrace CFSE cell proliferation dye (C34554, Molecular Probes, Eugene, OR) according to the manufacturer’s instructions. Briefly, CFSE stock solution (in DMSO, Fisher-Thermal Scientific) was diluted with PBS to a working solution of 0.2 μM. Cells were resuspended in pre-warmed CFSE working solution and incubated at 37 °C for 15 min in the incubator. Cells were pelleted by centrifugation, resuspended in pre-warmed (37 °C) RPMI medium and incubated at 37 °C for 30 min. Cells were then pelleted and resuspended in medium to a density of 4 × 10^5^ cells/ml. Labeled CT26 cells were transferred to each well of a U-bottom 96-well plate. CTLs were purified with the Lymphocytes Separation Medium, washed in medium and added to the tumor cell cultures at various ratios. The tumor-CTL mixtures were cultured in the CO2 incubator for approximately 24 h. Culture supernatant was collected. Adherent tumor cells were harvested using 0.05% Trypsin-EDTA solution and combined with the cultured supernatant. The collected tumor cell and CTL mixtures was pelleted by centrifugation, resuspended in PBS and stained with PI. Cells were analyzed immediately by flow cytometry. CFSE^+^ tumor cells were gated and analyzed for PI^+^ cells.

### *In vivo* toxicity assay

CT26 cells (2.5 × 10^5^ cells/mouse) were injected into tail vein of BALB/c mice intravenously. Eight days later, ceramide analogs were dissolved in DMSO and diluted with peanut oil such that DMSO concentration is less than 5%, and injected to the tumor-bearing mice intraperitoneally.

### Statistical analysis

Student’s *t* test was used to compare differences between different treatment groups. A *p* < 0.05 was taken as statistically significant.

## Additional Information

**How to cite this article**: Coe, G. L. *et al*. Ceramide mediates FasL-induced caspase 8 activation in colon carcinoma cells to enhance FasL-induced cytotoxicity by tumor-specific cytotoxic T lymphocytes. *Sci. Rep.* **6**, 30816; doi: 10.1038/srep30816 (2016).

## Supplementary Material

Supplementary Information
