# Supplementary material for: Ceramide mediates FasL-induced caspase 8 activation in colon carcinoma cells to enhance FasL-induced cytotoxicity by tumor-specific cytotoxic T lymphocytes
Source: Sci Rep. 2016 Aug 4;6:30816. doi: 10.1038/srep30816 (PMC4973238; doi:10.1038/srep30816)
Supplement: Supplementary Information [file srep30816-s1.pdf]

Supplemental Data

Ceramide mediates FasL-induced caspase 8 activation in colon carcinoma cells to enhance FasL-induced cytotoxicity by tumor-specific cytotoxic T lymphocytes

Genevieve L. Coe[1,2*], Priscilla S. Redd[1,2,3*], Amy V. Paschall[1,2,3], Chunwan Lu[1,3], Lilly Gu[1], Thomas Albers[4], Iryna O. Lebedyeva[4], and Kebin Liu[1,2,3]

[1]Department of Biochemistry and Molecular Biology, Medical College of Georgia, [2]Georgia Cancer Center, Augusta University, Augusta, GA 30912, USA. [3]Charlie Norwood VA Medical Center, Augusta, GA 30904. USA. [4]Department of Pharmaceutical & Biomedical Sciences, University of Georgia, Athens, GA 30602, USA. [5]Department of Chemistry and Physics, Augusta University, Augusta, GA 30912. USA.

***Equal contribution**

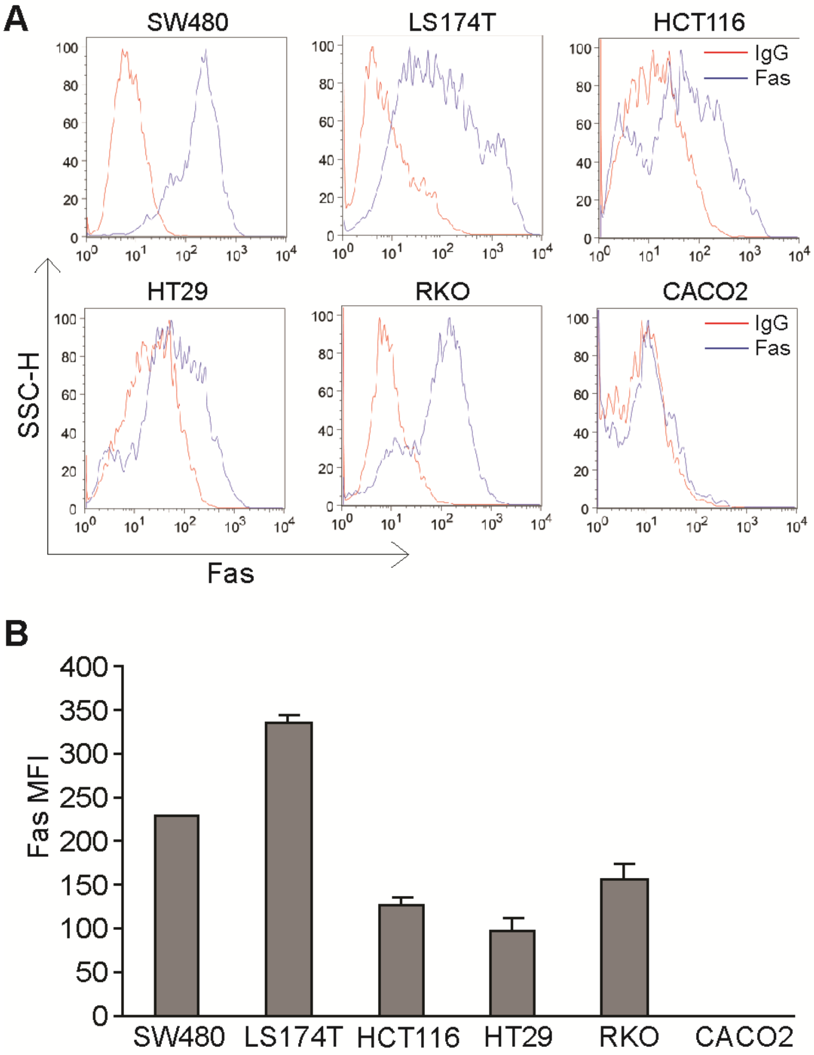

Figure S1. Fas protein level on human colon carcinoma cell surface. A. The indicated human colon carcinoma cells were stained with IgG isotype control or anti-human Fas mAbs and analyzed by flow cytometry. Shown are plots of each cell line. B. The mean fluorescent intensity (MFI) of Fas of each cell line is calculated and presented. Column: mean; Bar:SD.

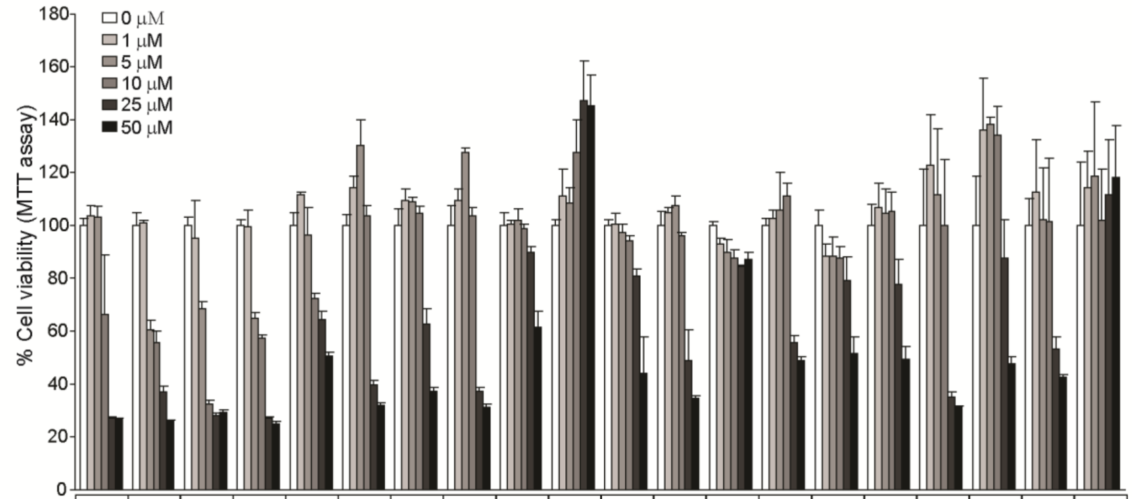

Analog	IG1	IG2	IG3	IG4	IG5	IG6	IG7	IG8	IG9	IG10	IG11	IG12	IG13	IG14	IG15	IG16	IG17	IG18	IG19	IG20
IC_{50} (μM)	10.0	16.5	5.3	6.3	9.2	13.5	18.4	13.7	29.4	ND	47.6	15.2	ND	19.3	27.3	20.0	12.9	20.0	15.6	ND

*ND: not determined

Figure S2

Figure S2. Cytotoxicity of ceramide analogs to human colon carcinoma cells. Human colon carcinoma SW480 cells were cultured in the presence of ceramide analogs at the indicated concentrations for three days. Cell viability was determined by MTT assays. % cell viability of control was set at 100% and cell viability of treatment groups was calculated as % of the control groups. IC_{50} was calculated using GraphPad Prism program.

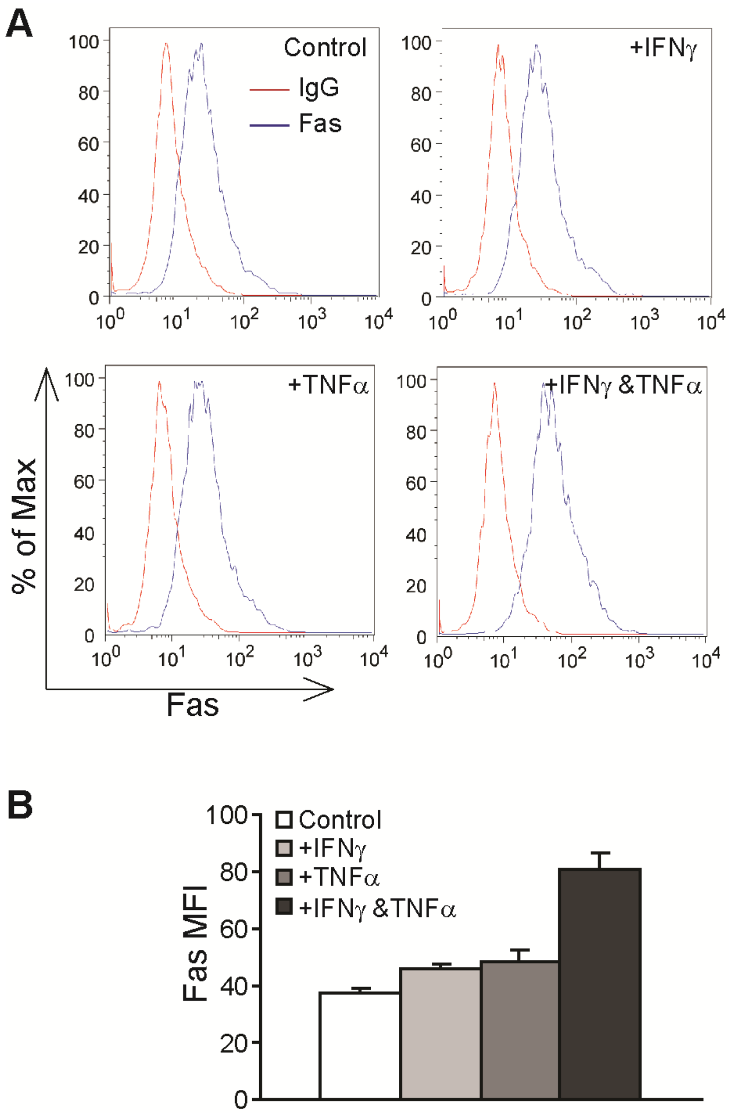

Figure S3. Fas protein level on mouse colon carcinoma cell surface. A. CT26 cells were cutured in the presence of IFNγ (100 U/ml), TNFα (100 U/ml), or both IFNγ and TNFα for approximately 24h. Cells were then stained with IgG isotype control or anti-mouse Fas mAbs and analyzed by flow cytometry. Shown are plots of each treatment group. **B**. The mean fluorescent intensity (MFI) of mouse Fas of each treatment group is calculated and presented. Column: mean; Bar:SD.

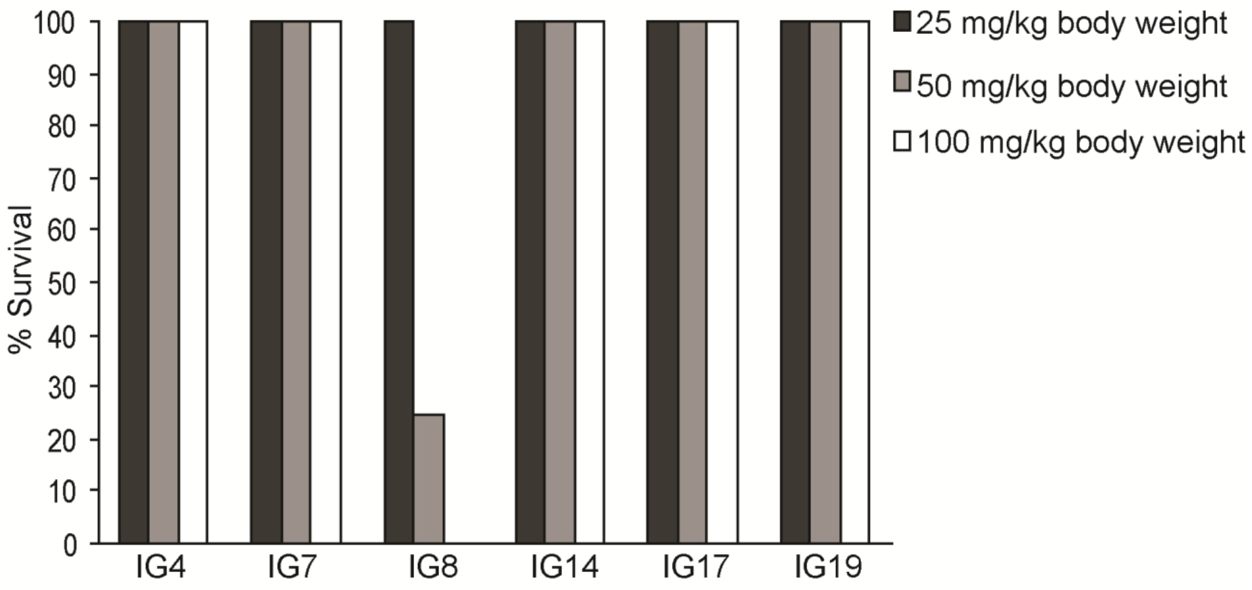

Figure S4. Ceramide analog toxicity. CT26 cells were injected to BALB/c mice intravenously. Eight days later, the indicated ceramide analogs at the indicated doses were injected to the tumor-bearing mice (n=3). Mouse survival was recorded and shown.

Table S1. Fas protein level in normal human colon and colon cancer tissues

	Normal Colon			Adenomas			Adenocarcinomas			LN Metastases			Liver metastases		
	H	M	L	H	M	L	H	M	L	H	M	L	H	M	L
*Fas protein level (%)	100<br>5/5	0<br>0/5	0<br>0/5	64<br>9/14	36<br>5/14	0<br>0/14	35.5<br>5/14	29<br>4/14	35.5<br>5/14	40<br>2/5	20<br>1/5	40<br>2/5	0<br>0/7	14<br>1/7	86<br>6/7

*H: high, M: medium, L: low to undetectable. The Fas protein level in normal human colon tissues is set as high and used as reference for scoring Fas protein level in the tumor tissues. The number under the percentage indicates number of specimens in that category vs total number of specimens.

Table S2. Ceramide analog compound structures

**IG1**<br>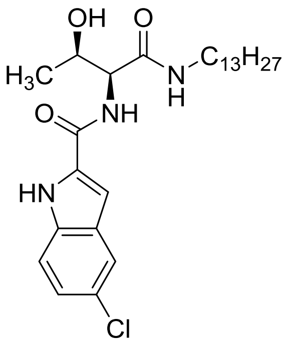<br>M. W.: 478.07	**IG2**<br>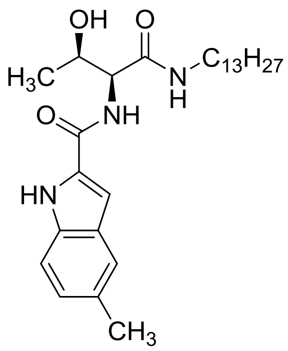<br>M. W.: 457.66	**IG3**<br>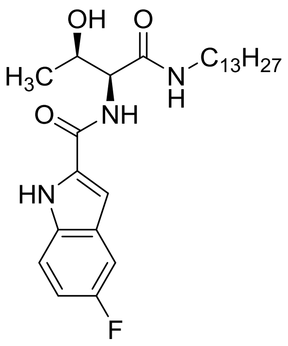<br>M. W.: 461.62	**IG4**<br>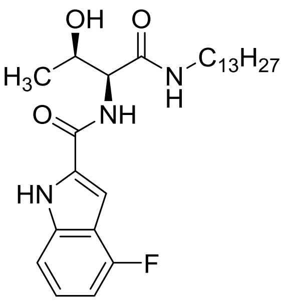<br>M. W.: 461.62
**IG5**<br>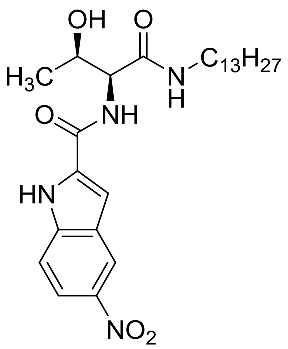<br>M. W.: 488.63	**IG6**<br>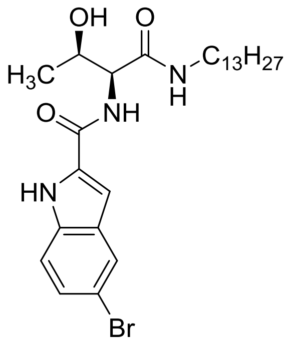<br>M. W.: 522.53	**IG7**<br>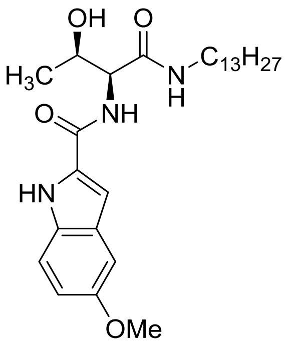<br>M. W.: 473.66	**IG8**<br>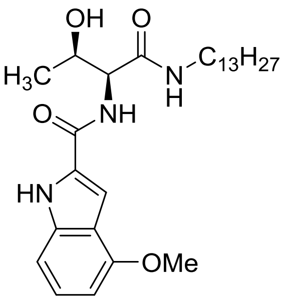<br>M. W.: 473.66
**IG9**<br>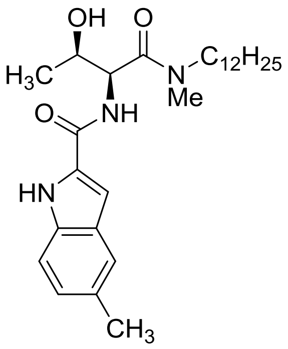<br>M. W.: 457.66	**IG10**<br>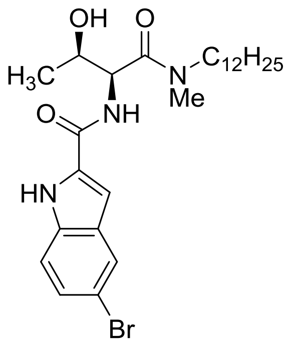<br>M. W.: 522.53	**IG11**<br>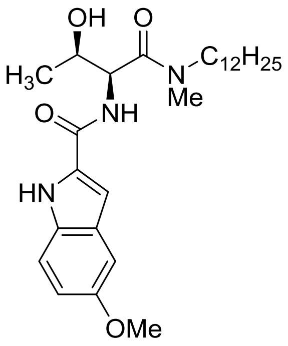<br>M. W.: 473.66	**IG12**<br>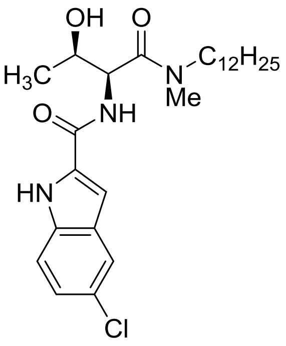<br>M. W.: 478.07

**IG13**<br>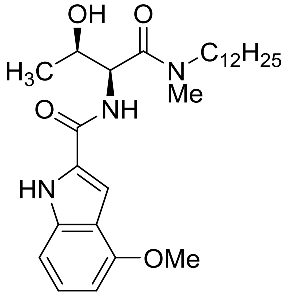<br>M. W.: 473.66	**IG14**<br>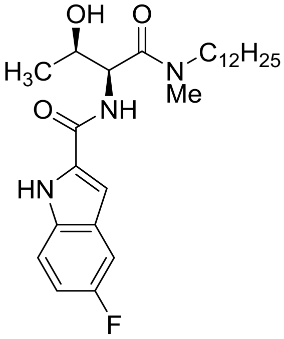<br>M. W.: 461.62	**IG15**<br>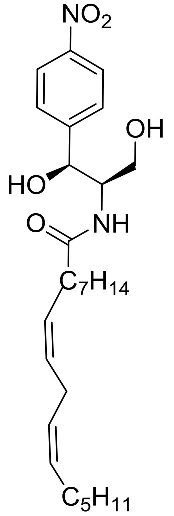<br>M.W.: 474.64	**IG16**<br>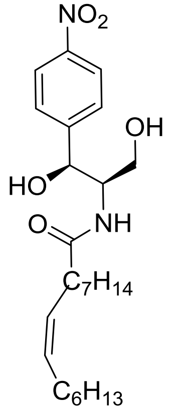<br>M.W.: 448.60
**IG17**<br>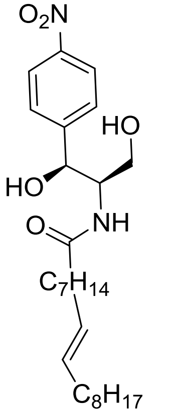<br>M.W.: 476.65	**IG18**<br>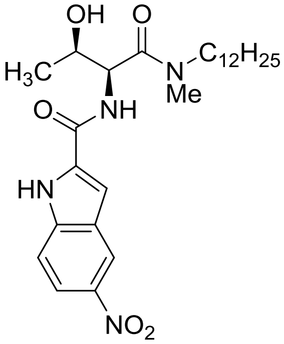<br>M. W.: 488.63	**IG19**<br>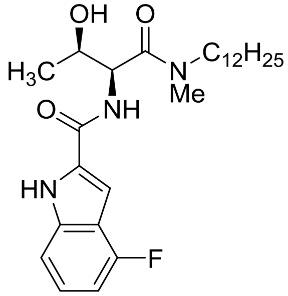<br>M. W.: 461.62	**IG20**<br>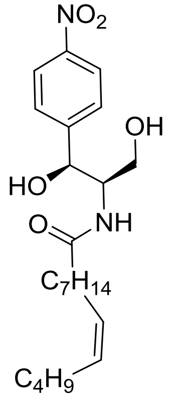<br>M.W.: 420.55
